# Host Cell Factors in Filovirus Entry: Novel Players, New Insights

**DOI:** 10.3390/v4123336

**Published:** 2012-11-26

**Authors:** Heike Hofmann-Winkler, Franziska Kaup, Stefan Pöhlmann

**Affiliations:** German Primate Center, Infection Biology Unit, Kellnerweg 4, 37077 Göttingen, Germany; Email: fkaup@dpz.eu

**Keywords:** filovirus, Ebola, Marburg, entry, cathepsins, TIM-1, Axl, NPC1

## Abstract

Filoviruses cause severe hemorrhagic fever in humans with high case-fatality rates. The cellular factors exploited by filoviruses for their spread constitute potential targets for intervention, but are incompletely defined. The viral glycoprotein (GP) mediates filovirus entry into host cells. Recent studies revealed important insights into the host cell molecules engaged by GP for cellular entry. The binding of GP to cellular lectins was found to concentrate virions onto susceptible cells and might contribute to the early and sustained infection of macrophages and dendritic cells, important viral targets. Tyrosine kinase receptors were shown to promote macropinocytic uptake of filoviruses into a subset of susceptible cells without binding to GP, while interactions between GP and human T cell Ig mucin 1 (TIM-1) might contribute to filovirus infection of mucosal epithelial cells. Moreover, GP engagement of the cholesterol transporter Niemann-Pick C1 was demonstrated to be essential for GP-mediated fusion of the viral envelope with a host cell membrane. Finally, mutagenic and structural analyses defined GP domains which interact with these host cell factors. Here, we will review the recent progress in elucidating the molecular interactions underlying filovirus entry and discuss their implications for our understanding of the viral cell tropism.

## 1. Introduction

Filovirus infection causes severe hemorrhagic fever in humans and non-human primates. Outbreaks of filovirus hemorrhagic fever occur in equatorial Africa and are associated with high case-fatality rates [1]. At present, neither vaccines nor antiviral drugs have been approved for combating filovirus infection. The *filoviridae* family comprises two genera, *Ebolavirus* (the ebolaviruses) and *Marburgvirus* (the marburgviruses). The genus *Marburgvirus* includes a single species, *Marburg marburgvirus*, which has two members, Marburg virus (MARV) and Ravn virus (RAVV). The genus *Ebolavirus* includes five species, each of which has a single member: *Zaire ebolavirus* (Ebola virus, EBOV), *Sudan ebolavirus* (Sudan virus, SUDV), *Taï Forest ebolavirus* (Taï Forest virus, TAFV), *Bundibugyo ebolavirus* (Bundibugyo virus, BDBV) and *Reston ebolavirus* (Reston virus, RESTV) [2,3]. Filoviruses exhibit different virulence in humans: EBOV and MARV infection is associated with case‑fatality rates of up to 90% [4,5] while RESTV seems to be apathogenic [6,7,8]. Nevertheless, infection of non-human primates with RESTV can induce hemorrhagic fever [9]. Evidence is emerging that African [10,11,12,13], Asian [14] and possibly also European [15] bats are natural reservoirs of filoviruses and these animals could transmit the virus directly to humans or via intermediate hosts, including gorillas [16,17] and swine [6,14,18]. Thus, filoviruses pose a threat to human and animal health in different continents, but virulence factors and pathogenesis are incompletely understood.

Deciphering filovirus pathogenesis requires the elucidation of filovirus interactions with host cells. The entry of filoviruses into target cells is the first essential step in the viral life cycle, and the viral and cellular factors involved in this process are potential targets for antiviral strategies. Infectious filovirus entry is mediated by the viral glycoprotein (GP), which is the only viral envelope protein and thus constitutes the sole target for the neutralizing antibody response. Consequently, defining which domains in GP are essential for cellular entry and can be targeted by the humoral immune response is pivotal to the design of effective vaccines. In addition, insights into the multiple, sequential interactions of GP with host cell factors required for cellular entry can define novel targets for therapeutic inhibition. Key components of the filovirus entry cascade have been identified in the recent years, and some of these discoveries have already been translated into novel antiviral approaches in cell culture and small animal models [19,20,21,22,23,24]. Here, we will review the current knowledge of the host cell factors involved in the cellular entry of filoviruses and we will discuss the implications of key findings in this area for our understanding of viral pathogenesis.

## 2. The Filoviral Glycoprotein: Structure and Function

The filovirus particle is predominantly of filamentous nature and consists of seven structural proteins and a non-segmented, single-stranded RNA genome of negative polarity. In the viral envelope, a single type I transmembrane glycoprotein is incorporated, which is a virulence factor [25] and the sole viral determinant of host cell entry. Thus, GP mediates attachment of virions to host cells and fusion of the viral envelope with a host cell membrane, which is essential for delivery of viral proteins and nucleic acid into the host cell cytoplasm [26,27,28,29,30]. To facilitate virus entry, GP interacts with host cell factors, and the expression pattern of these factors is believed to be a central determinant of filovirus cell tropism. In addition, the various soluble forms of GP produced in the context of ebolavirus infection might modulate viral pathogenicity, as reviewed elsewhere [31].

### 2.1. Biosynthesis and Posttranslational Modification of the Filovirus Glycoprotein

The filoviral GP is synthesized as a precursor protein, GP_0_, in the secretory pathway of infected cells. Upon transport of GP_0_ into the Golgi apparatus, subtilisin-like proprotein convertases, in particular furin, process the precursor protein into two subunits, the surface unit GP_1_ and the transmembrane unit GP_2_ [32]. Both subunits remain covalently linked by an intermolecular disulfide bond and trimers of GP_1_-GP_2_ heterodimers (GP_1_,_2_) are inserted into the cellular and the viral membrane [33]. The filoviral GP_1,2_ is heavily glycosylated and harbors numerous consensus sites for N-linked glycosylation and GP_1_ glycosylation has been determined on the molecular level [34,35]. In addition, a mucin-like region (MLR) at the C-terminus of GP_1_, which is highly variable among filovirus species [28,33,36,37,38], is extensively modified by O-linked glycans. The MLR is dispensable for entry into cell lines, as lentiviral vectors (pseudotypes) bearing GP_1,2_ mutants with a deleted MLR show comparable or even enhanced capacity to transduce certain cell lines (for instance Vero cells) compared to particles carrying wild type GP_1,2_ [28,36,39,40,41]. However, the MLR seems to be important for association with several host cell lectins, as discussed below, and might have an important role in immune evasion and filovirus pathogenesis.

### 2.2. The Filovirus Glycoprotein Is a Class I Membrane Fusion Protein

**Figure 1:**
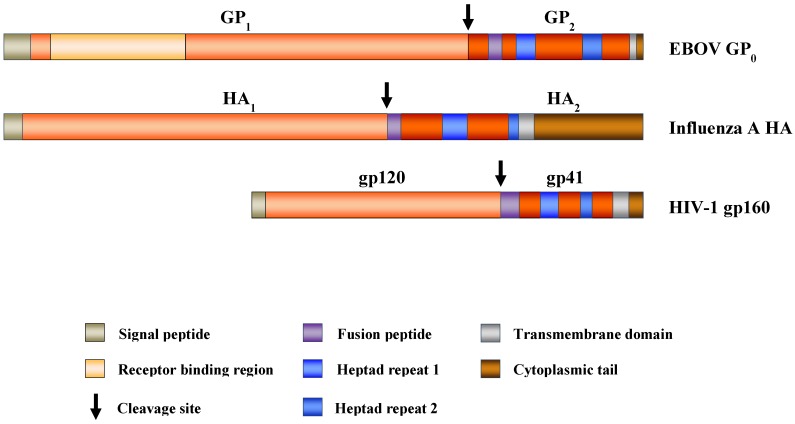
Domain organization of selected viral class I membrane fusion proteins. Viral class I membrane fusion proteins contain an N-terminal surface unit, which binds to cellular receptors, and a C-terminal transmembrane unit, which harbors the membrane fusion machinery. The glycoproteins are synthesized as inactive precursor proteins, which are primed for membrane fusion by proteolytic processing at the border between both subunits (the activating cleavage site is indicated by an arrow). For Ebola virus (EBOV)- viral glycoprotein (GP_0_), the receptor binding region (RBR) is shown. The C-terminal membrane fusion subunits of the respective glycoproteins, which anchor the proteins in the viral membranes, contain the following characteristic architectural elements required for membrane fusion: heptad repeats and a hydrophobic fusion peptide. The figure is adapted from [44,54].

Mutagenic analyses and resolution of the structure of GP_1,2_ at the atomic level identified features also present in other viral glycoproteins, termed class I membrane fusion proteins (Figure 1), including the human immunodeficiency viruses (HIV) envelope protein and the influenza viruses hemagglutinin [37,42,43]. Thus, class I membrane fusion proteins form metastable trimers which are oriented perpendicular to the viral membrane. Each monomer consist of an N-terminal surface subunit, which contains a receptor-binding domain, and a C-terminal transmembrane unit inserted in the viral membrane, which harbors domains integral to the membrane fusion reaction, a hydrophobic fusion peptide or fusion loop and two heptad repeats (HR). The surface and transmembrane units are separated by a proteolytic cleavage site, and processing of this site by a host cell protease primes the proteins for membrane fusion. The membrane fusion involves a major conformation rearrangement of the transmembrane unit, resulting in the formation of a characteristic, highly stable trimer-of-hairpins with a central α-helical coiled-coil [44].

As predicted for class I membrane fusion proteins, the surface subunit, GP_1_, of the filoviral GP contains a receptor binding region (RBR), which facilitates GP binding to host cells [26,27,28,29,30]. The RBR localizes to the N-terminus of GP_1_ and is relatively conserved between EBOV- and MARV-GP (47% amino acid identity) which are believed to share a cellular receptor [36,45]. GP_1_ and GP_2_ are separated by a protease sensitive site, which is cleaved by subtilisin-like proprotein convertases, in particular furin. However, in contrast to other class I membrane fusion proteins, the furin consensus site is dispensable for cellular entry [46,47] and priming of GP is facilitated by endosomal cathepsins, as discussed below. The transmembrane unit GP_2_ contains an N-terminal fusion loop and two HRs, which are conserved between the GP_2 _subunits of ebola- and marburgviruses. In addition, a synthetic peptide corresponding to the C-terminal HR sequence of EBOV-GP_2_ inhibited both EBOV and MARV infection [19], indicating that both viruses use similar strategies to facilitate membrane fusion [39,48,49,50,51,52,53]. The membrane fusion reaction results in the formation of a thermostable trimer-of-hairpins structure [42,43], as expected for a class I membrane fusion protein.

### 2.3. Filovirus Fusion with Host Cell Membranes

In the native, pre-fusion form of GP_1,2_, the N-terminal HR is hidden in the GP_1_-GP_2_ trimeric structure by the RBR [37], which is protected by a glycan cap, whereas the fusion loop curls around the outside of GP_1_ [37,45,55]. Priming of GP_1,2_ for membrane fusion occurs in host cell endosomes, where pH-dependent proteases remove the glycan cap [56,57], which in turn leads to the exposure of the fusion loop and allows its insertion into the host cell membrane [37,58]. Thereafter, HR1 and HR2 fold back onto each other, resulting in the formation of a thermostable trimer-of-hairpin structure [42,43,59], which brings viral and target cell membranes into close vicinity and ultimately facilitates membrane fusion. Several host cell factors are involved in priming and triggering of filovirus GP_1,2_ for membrane fusion and will be discussed in detail below.

## 3. Cell and Organ Tropism of Filoviruses

The cellular molecules involved in filovirus entry govern the spectrum of cells susceptible to filovirus infection. Before we discuss GP_1,2_ interactions with these factors, we will therefore provide a brief overview of the filovirus cell tropism. Filoviruses exhibit a broad cell and organ tropism in infected humans and non-human primates. However, at the early stages of the infection, cells of the mononuclear phagocyte system are mainly targeted, in particular macrophages and dendritic cells in the spleen, lymph nodes and liver [60,61,62,63,64,65,66,67,68,69,70]. Infection of these cells not only amplifies the virus [71] and ensures its rapid dissemination [72], but also triggers the uncontrolled release of pro-inflammatory cytokines [60,66,70], a hallmark of filovirus pathogenesis.

Secondary targets of filovirus infection are mainly fibroblasts and endothelial cells located in many different organs, including liver, kidney and testis, and these cells are permissive to robust, lytic viral replication [64,73]. It has been proposed that ebolavirus infection of the vascular endothelium and the ensuing GP_1,2_-induced cell rounding might result in loss of vascular integrity and hemorrhage [28]. However, the vascular endothelium is a late target in EBOV infected non-human primates, and no obvious cytotoxic effects are associated with its infection [64], suggesting that hemorrhage is not a direct consequence of ebolavirus infection of vascular endothelial cells. Apart from fibroblasts and endothelial cells, virtually all cell types are susceptible to filovirus infection, and infectious EBOV and MARV could be isolated from all tissues tested [64,67,74,75,76,77,78]. The only cells refractory to the otherwise pantropic filoviruses are lymphocytes [65,67], and experiments with pseudotypes showed that these cells are not susceptible to GP_1,2_-driven host cell entry, potentially because of lack of host cell factors required for appropriate uptake and intracellular trafficking [26,27,27,79,80,81,82,83,84].

## 4. Host Cell Factors Promoting Infectious Filovirus Entry

**Figure 2:**
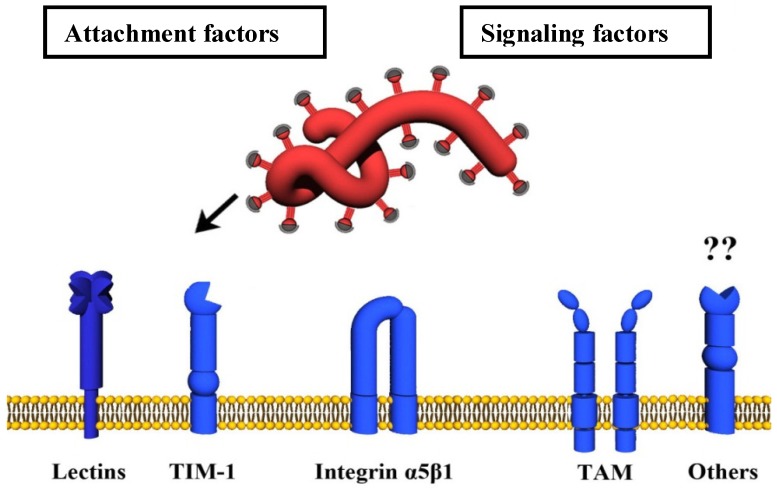
Host cell surface proteins involved in filovirus uptake. Cellular lectins bind glycans present on the filoviral GP_1,2_ and thereby promote attachment to target cells. The surface molecule TIM-1 interacts with GP_1,2_ inserted in the viral membrane. TAM proteins do not bind to GP_1,2_ but elicit signals which promote viral uptake by macropinocytosis. Integrins might interact with GP_1,2_ and are important for intracellular processing of GP_1,2_.

**Figure 3:**
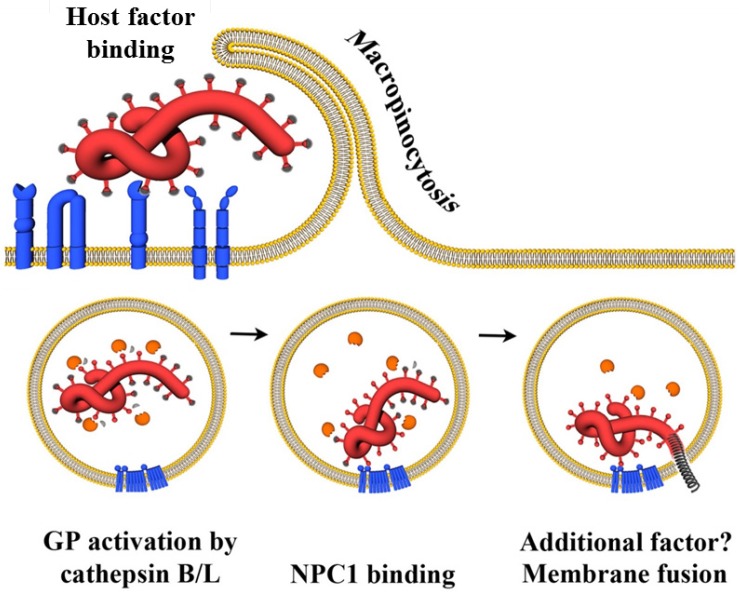
Infectious entry of filoviruses into target cells. After interaction between GP_1,2_ and cellular surface molecules, virions are internalized via macropinocytosis into the endosomal compartment. Subsequently, the endosomal cysteine proteases cathepsin B and L proteolytically process GP_1,2 _, thereby removing the glycan cap (indicated by grey caps) and allowing primed GP_1_ binding to Niemann-Pick C1 (NPC1), which is essential for the following virus-host membrane fusion process. Finally, a so far incompletely understood stimulus triggers the membrane fusion activity in GP_2_.

The entry of enveloped viruses into host cells commences with the attachment of the virus to the cell surface, which is frequently promoted by relatively nonspecific interactions between the viral GP and cellular attachment-promoting factors. Subsequently, highly specific engagement of cellular molecules by the viral GP is essential to trigger uptake of virions into target cells and/or fusion of the viral with a host cell membrane. A virus entry receptor is usually defined as a cellular binding partner of a viral GP which is essential for infectious viral entry into host cells. Given the complexity of filovirus entry, which involves both cell surface molecules and intracellular proteins, only some of which physically interact with GP, we will not employ the classical receptor definition in our description of filovirus entry. Instead, discriminate between attachment factors, which interact with GP_1,2_ at the cellular membrane and promote viral attachment to cells, signaling factors, which induce filovirus uptake through activating signaling cascades (Figure 2), and endo-/lysosomal host factors, which prime and activate GP_1,2_ for membrane fusion (Figure 3).

### 4.1. Attachment Factors

#### 4.1.1. Carbohydrate-Binding Host Cell Factors (Lectins)

The cellular lectins DC-SIGN, DC-SIGNR, LSECtin, ASPGR-1 and hMGL [41,85,86,87,88,89] can promote filovirus entry into transfected cells (Table 1). An initial study suggested that expression of some of these lectins might be sufficient to render target cells susceptible to filovirus infection [85]. However, subsequent work showed that lectins merely facilitate usage of low levels of so far unidentified molecules important for filovirus entry [81,90,91]. Augmentation of filovirus infection by lectins depends on binding of these proteins to glycans on GP_1,2_, and the respective lectins exhibit differences in their carbohydrate specificities, which are reflected by differential requirements for GP_1,2_ binding. Thus, the extensively O-glycosylated MLR is dispensable for GP_1,2_ binding to DC‑SIGN and DC‑SIGNR [92], which mainly recognize high-mannose glycans, while deletion of the MLR impedes interactions with hMGL, which recognizes terminal galactose-/N-acetylgalactosamine [41,50,93]. In addition, differences in lectin engagement among the ebolavirus species and MARV have been noted [41,92]. The expression of the GP-binding lectins on relevant filovirus target cells, including tissue macrophages and dendritic cells (hMGL, DC-SIGN) [94,95], hepatocytes (ASGPR-1) [96] and liver and lymph node sinusoidal endothelial cells (LSECtin, DC-SIGNR) [97,98] suggests that they could modulate filovirus spread in the infected host. However, there is currently little evidence that endogenous lectin expression appreciably augments filovirus entry into primary target cells [90], and the role of lectins in filovirus spread in animal models has not been examined. In the context of HIV-1 infection, it has been demonstrated that viral binding to DC-SIGN on dendritic cells triggers signal transduction and thereby commandeers the cell to undergo aberrant maturation and to produce immunosuppressive cytokines [99], which is believed to promote viral spread. Whether a similar mechanism is operative during filovirus infection is unknown. Finally, it is noteworthy that secreted lectins can modulate filovirus infection. Thus, recombinant mannose-binding C-type lectin (MBL) was shown to protect mice from a lethal EBOV infection potentially by targeting the virus for phagocytosis and complement-directed lysis [100].

**Table 1:** Overview and expression of lectins involved in filovirus attachment.

GP-binding lectins	Tissue distribution	Reference
ASGPR-1	hepatocytes	[86]
hMGL	monocyte-derived immature dendritic cells intermediate precursors of macrophages	[41]
DC-SIGN	dendritic cells fetal macrophages decidual macrophages alveolar macrophages platelets activated primary B-cells	[85,85,88,90,101,102,103]
DC-SIGNR	endothelial cells in the placenta endothelial cells in the liver and lymph node sinuses	[85]
LSECtin	sinusoidal endothelial cells in liver, lymph nodes and bone marrow thymic dendritic cells monocyte-derived macrophages peripheral blood cells	[34,87,102]

#### 4.1.2. TIM-1

The human T cell Ig mucin 1 (TIM-1) surface molecule was initially found to be a cellular receptor for hepatitis A virus [104]. By a bioinformatics-based correlation analysis between gene expression proﬁles and susceptibility of cell lines to EBOV-GP_1,2_-driven infection, TIM-1 was recently also identified as an entry factor for filoviruses [105]. TIM-1 is a type I membrane glycoprotein with an extracellular IgV domain and a mucin-like domain predicted to be heavily O-glycosylated [106]. The IgV domain allows highly specific recognition of phosphatidylserine exposed on the surface of apoptotic cells, and TIM-1 was shown to be involved in the clearance of apoptotic cells [107,108]. TIM-1 is expressed on activated T-cells, epithelial cells, conjunctiva and renal tissue [105,109,110] as well as certain cell lines including the liver cell line Huh7 [105] (Table 2), which is permissive for filovirus replication [111].

A physical interaction between the receptor binding domain of EBOV-GP_1_ and soluble TIM-1 could be demonstrated [105], and soluble TIM-1 was shown to block EBOV-GP_1,2_-driven infection, indicating that TIM-1-dependent filovirus entry requires GP_1,2_ interactions with this protein. RNA knock-down of endogenous TIM-1 inhibited pseudovirus entry into otherwise susceptible cell lines, and ectopic expression of TIM-1 on TIM-1-negative cell lines enhanced infection [105], indicating that TIM-1 indeed promotes filovirus infection of certain cell lines. However, macrophages and dendritic cells, which are important targets of filovirus infection, lack TIM-1 expression [110], suggesting that so far unidentified cellular factors facilitate filovirus entry into these cells. Finally, it would be interesting to determine whether TIM-1 intracellular signaling [112,113] might contribute to filovirus GP-mediated cellular uptake.

**Table 2:** Overview of host cell factors involved in filovirus entry.

Host factor	Expression	Analysis in cell lines	Reference
TIM-1	Epithelia of conjunctiva and cornea Injured kidney tubuli epithelia Activated Th2 cells Human airway epithelia Mast cells	786-O A498 ACHN CAKI-1 TK-10 UO-31 RXF393 Huh7 Vero E6 293T-H3	[105,109,110]
Axl	broad expression in cells of the mature immune, nervous, reproductive and vascular systems	Vero E6 HT1080 293 HeLa Cos7 A549 SNB19 HuVECs SN12C HFF	[114,115,116,117,118,119,120]
Integrin αV β1	Ubiquitous	Hela HepG2 Hep3B	[121,122,123,124]
Cathepsin B and L	Ubiquitous	Vero E6 MEF 293T	[24,56,57,125,126,127,128]
NPC1	Ubiquitous	HUVEC Vero E6 Cloned HAP-1 others	[20,129,130]

### 4.2. Signaling Factors

#### 4.2.1. TAM Family of Tyrosine Kinase Receptors

Another interesting group of molecules shown to be involved in ebolavirus infectious entry are members of the Tyro3/Axl/Mer (TAM) family of receptor tyrosine kinases [119,131]. These proteins contain a kinase domain and an adhesion-molecule-like extracellular domain and play important roles in diverse biological processes including cell proliferation and survival, cell adhesion and cytokine release [131]. Members of the TAM family are evolutionarily conserved between vertebrate species and the respective proteins are widely expressed in adult tissues, most prominently in the brain, lung, kidney, lymphatic tissue and the vascular system [118,119] (Table 2). The natural ligands for TAM receptors are the structurally homologous proteins Gas6 and protein S [132,133]. These secreted proteins bind to phosphatidylserine residues exposed by apoptotic cells [134,135], and ligand-activated TAM receptors have been found to inhibit inflammation pathways in macrophages and dendritic cells [120].

By introduction of a cDNA library derived from permissive VeroE6 cells into non-susceptible Jurkat lymphocytes, Axl was identified as an EBOV entry factor expressed at the cell surface [117]. The ectopic expression of Axl and the TAM family members DTK and MER on lymphoid cells allowed for transduction of GP_1,2_-harboring pseudotypes, which could be blocked by Axl-specific antibodies, confirming that TAM-proteins could promote filovirus entry [117], although the mechanism underlying the antibody-mediated blockade of viral entry is currently unclear. Mutational analysis revealed that both the extracellular ligand binding domain and the cytoplasmic tail of Axl were required for efficient GP_1,2_-mediated entry [116,117]. A role of TAM family proteins in filovirus entry was confirmed by an independent study, which showed that expression of an mRNA encoding for Axl correlates with susceptibility of cell lines to EBOV infection [114]. The use of TAM protein‑specific antibodies and siRNA knockdown indeed identified several cell lines, in which EBOV-GP_1,2_-faciliated entry was dependent on Axl expression. In contrast, down-regulation of Axl‑expression in other cell lines did not compromise GP_1,2_-driven entry [114,115,117]. So far, all studies failed to detect a direct interaction between EBOV-GP_1_ and Axl. However, Axl expression was shown to augment internalization of EBOV-GP_1,2_-bearing pseudovirions and virus-cell fusion [114], and this activity correlated with enhanced macropinocytosis in Axl-expressing cells [115]. Thus, Axl might promote filovirus uptake by macropinocytosis, which was previously shown to be a pathway exploited by filoviruses for cellular uptake [136,137]. Indeed, inhibition of Axl and blockade of PIK3, which is important for macropinocytosis and Axl-dependent signaling, both inhibit filovirus entry in a cell type-dependent fashion [21,125]. Finally, it is noteworthy that Gas6 can promote Sindbis virus entry by bridging phosphatidylserine present in the viral envelope to Axl localized on target cells [138]. Whether a similar mechanism operates in the context of filovirus entry remains to be investigated. The observation that recombinant Gas6 inhibits Axl- and DTK-dependent GP_1,2_-mediated entry [117] might argue against this hypothesis.

#### 4.2.2. α5β1-Integrin

Integrins are cell surface expressed heterodimeric type I transmembrane glycoproteins, which are composed of two non-covalently linked subunits (α and β) [122]. The integrin family in mammals comprises eighteen α and eight β subunits which can assemble into 24 different heterodimers [139]. These heterodimers, in turn, convey specificity to cell-cell and cell-extracellular matrix adhesion, immune cell recruitment, extravasation, and signaling events [121,140]. Members of the integrin family have been discovered as attachment factors or receptors for a large number of enveloped and non-enveloped viruses, including herpesviruses [141,142,143], adenoviruses [144,145], hantaviruses [146,147,148,149], picornaviruses [150,151,152], and reoviruses [153,154]. The association with viral surface proteins and microbial pathogens are followed by multiple signaling events, some of which promote cytoskeletal reorganization and thus facilitate receptor-mediated endocytosis (as reviewed in [155,156] and references therein). It is not surprising that numerous viruses hijack integrins for infectious entry, as they are widely expressed in various tissues throughout the body (Table 2).

Expression of EBOV-GP_1,2_ was initially shown to interfere with surface expression of various cellular membrane proteins, including α3 and ß1 integrins [40,124]. However, a more recent study suggested that GP_1,2_ expression does not reduce cell surface levels of integrins but rather sterically occludes epitopes in these proteins otherwise recognized by antibodies [157]. Experiments with EBOV-GP_1,2_-bearing pseudotypes demonstrated that soluble recombinant ß1 integrin or ß1-reactive antibodies diminish GP_1,2_ driven entry, suggesting that GP_1,2_ might need to engage ß1 integrins for infectious entry [124]. However, a direct interaction between EBOV-GP_1,2_ and integrins remains to be demonstrated. Work by Schornberg and colleagues provided evidence that α5β1-integrin is required for expression of the double chain forms of cathepsin B and L and for full cathepsin L activity [123], an endosomal protease involved in priming of GP_1,2_ for membrane fusion, as discussed below. In contrast, α5β1-integrin was dispensable for GP_1,2_-mediated binding and uptake into target cells [123]. These observations suggest that α5β1-integrin indirectly promotes GP_1,2_-driven entry by ensuring activity of GP_1,2_-priming cysteine proteases or by stimulating the protease maturation pathway, which might be required for viral entry. Whether physical interactions of GP_1,2_ with α5β1-integrin also contribute to filovirus entry remains to be determined.

### 4.3. Endo-/Lysosomal Host Cell Factors

The membrane fusion reaction driven by the GPs of enveloped viruses can be triggered by several stimuli. For some viruses, engagement of a receptor at the cell surface activates fusion with the plasma membrane at neutral pH [158]. Alternatively, receptor binding resulting in virus internalization, and membrane fusion is stimulated by protonation of the viral GP in the acidic environment of the endosome [158]. The mild pH (6.5–6) of early endosomes is sufficient to trigger membrane fusion facilitated by the Nipah and Hendra virus GPs [159,160], while the low pH (5.5–4) environment of late endosomes and/or lysosomes activates the membrane fusion proteins of influenza, bunya and dengue viruses [161,162,163]. In addition, some viruses require both low pH and receptor engagement as triggers for membrane fusion [164], while others are triggered upon receptor engagement at the cell surface, but ultimately fuse with the endosomal membrane [165], indicating that complex determinants govern the nature of the trigger and the subcellular location of membrane fusion reaction.

Many viral GPs are synthesized as inactive precursor proteins which transit into a membrane fusion-competent state only upon proteolytic cleavage by host cell proteases, a process termed priming. A prominent example is the influenza virus, which depends on cleavage of its hemagglutinin by host cell proteases for acquisition of infectivity, and the nature of the cleavage sequences in HA determines the virulence of avian influenza viruses [166]. The subtilisin-like proprotein convertase furin is responsible for priming of several viral GPs in the Golgi apparatus of infected cells, and furin consensus sites are present in GP_0_ of all filoviruses excluding RESTV, which harbors an incomplete furin recognition site [32]. Despite of its conservation, several studies indicate that this motive is dispensable for filoviral spread in cell culture and infected animals [46,47], and the reason for its presence is unknown.

It has long been noticed that filoviruses depend on low pH for infectious cellular entry [26]. However, it has also been demonstrated that low pH does not trigger the fusion activity of GP_1,2_ [167]. This conundrum has been resolved by a study demonstrating that filoviruses are activated by endo‑/lysosomal cysteine proteases, which require a low pH environment for their enzymatic activity [57].

#### 4.3.1. Cathepsins B and L

Cathepsins comprise serine, aspartic and cysteine proteases and carry out diverse biological functions, including antigen processing for MHCII presentation [168,169,170]. Cysteine proteases of the papain family, some of which are localized in endosomes, are expressed as preproenzymes and are activated by proteolysis in the endoplasmatic reticulum and the late endosome/lysosome [168,169,170]. The low pH environment present in the latter compartment is essential for cathepsin enzymatic activity.

Chandran and colleagues demonstrated that two lysosomal cathepsins, cathepsin B and L, cleave filovirus GP_1,2_ and that cathepsin activity is essential for GP_1,2_-driven host cell entry [57]. This report showed that cathepsins B and L prime the filovirus GP_1,2_ for membrane fusion and proposed that cathepsin cleavage of virion-associated GP_1,2_ occurs in a sequential fashion: First, cathepsin L and/or B cleave GP_1,2_ into an 18 kDa form, which is fully infectious but still requires cathepsin B activity for infectious entry. Subsequently, the 18 kDa form is processed by cathepsin B and cleavage might be sufficient to trigger membrane fusion [57]. An alternative model for GP_1,2_ activation has been proposed by subsequent studies. Thus, Schornberg and colleagues demonstrated that processing of virion-inserted GP_1,2_ by recombinant cathepsin B and L or the bacterial protease thermolysin yielded a 19 kDa form of GP_1_ and was associated with a notable increase in infectivity [56], a finding confirmed by others [126]. Processing of GP_1_ into the 19 kDa from proceeded via 50 kDa and 20 kDa intermediates and its was speculated that the 20 kDa form might differ from the 18 kDa form observed by Chandran and colleagues only in the presence of a N-linked glycan [56]. Virions bearing the 19 kDa form were largely resistant to cathepsin B but not L inhibitors but remained sensitive to a lysosomotropic agent and a cysteine protease inhibitor. On the basis of these findings, a two-step model was proposed, suggesting that GP_1,2_ must first be processed by cathepsins B and L before the activity of a third lysosomal factor, potentially a thiol reductase, triggers GP_1,2_-dependent membrane fusion [56,171]. Indeed, subsequent studies provided evidence that the 19 kDa form represents a metastable conformation in which the fusion machinery is not yet exposed [37,172,173,174] and which can be triggered for membrane fusion by low pH and reduction [173]. In addition, it was demonstrated that cleavage of GP_1,2_ removes a glycan cap and the MLR, while the N-terminal RBR and GP_2_ remain in the molecule [172,175,176]. In sum, proteolytic processing by cathepsins B and L primes GP_1,2 _for membrane fusion and exposes the RBR. Subsequently, an incompletely understood stimulus triggers membrane fusion and these final steps of the lysosomal escape of filoviruses critically depend on GP_1,2_ binding to NPC1 (Figure 3), as discussed below.

Despite the importance of cathepsin B and L in priming EBOV-GP_1,2_ for membrane fusion in several cell lines, the dependence on these particular proteases for viral entry is not universal among filoviruses. A requirement for cathepsin B activity during entry of EBOV-, TAFV- and BDBV-but not SUDV-, RESTV and MARV-GP-bearing pseudotypes has been described, and the same group showed that particles harboring the GP_1,2_ of EBOV, SUDV and MARV exhibited enhanced transduction efficiency when cathepsin L was active in concert with cathepsin B. In contrast, entry of RESTV was dependent on a cysteine protease distinct from cathepsins B and L [125]. Furthermore, it has been reported that cathepsin L activity is dispensable for ebolavirus GP_1,2_-driven entry into Vero cells and mouse embryonic fibroblasts [57,125] as well as human monocyte-derived dendritic cells [128]. Moreover, the observation of the failure of EBOV-GP_1,2_-bearing pseudotypes to transduce CatB^−^/_−_ CatL^−^/_−_ mouse embryonic fibroblasts can be overcome by ectopic expression of CatB, suggests that a protease other than CatL is required for a post-CatB cleavage step necessary for membrane fusion [125]. In addition, transduction of primary human macrophages by EBOV-GP_1,2_-carrying pseudotypes was shown to be dependent on both cathepsin B and L, whereas MARV-GP_1,2_-facilitated entry was not blocked efficiently by cathepsin B/L inhibitors, suggesting that MARV-GP_1,2_ might employ a so far unknown protease for priming in macrophages [177]. It is also noteworthy that many studies investigating the role of cathepsins in filovirus host cell entry were performed with GP_1,2_‑bearing vectors and not with authentic filoviruses. It would thus be interesting to examine the effect of cathepsin B and L knock-out on filoviral spread and pathogenicity, particularly in the light of efforts to develop cathepsin inhibitors as treatment for SARS-coronavirus [178] and filovirus infection. The respective knock-out mice required for such studies have been described [179,180,181]. A study by Wong and colleagues indicates that blockade of cathepsin activity might result in the development of resistant viruses. Resistance was associated with mutations at the border between GP_1_ and GP_2_, which increased susceptibility to proteolytic cleavage and allowed GP_1,2_ priming by cysteine cathepsins other than cathepsin B and L [182]. Finally, it is noteworthy that expression of integrins previously suggested to promote filovirus entry is required for activity of cathepsin B and L [123], indicating an intricate interplay between some of the host cell molecules exploited by filoviruses for cellular entry.

#### 4.3.2. NPC1

An endosomal factor required for filovirus entry after GP_1,2 _priming by cathepsins has recently been discovered by two independent studies as the Niemann-Pick C1 (NPC1) protein. Cote and colleagues discovered that filoviruses depend on NPC1 for cellular entry by screening a library of chemical compounds for entry inhibitors [20], while Carette and coworkers found in a screen of haploid human cells that mutations in NPC1 are not compatible with filovirus GP_1,2_-driven entry [129]. Finally, a recent study found that CHO cells selected for resistance to EBOV-GP_1,2_-dependent entry harbored a defect in the *NPC1* gene [183]. The NPC1 protein is highly conserved among species and is ubiquitously expressed in human tissues, with the highest expression in the liver [184,185] (Table 2). The protein is an integral membrane protein of late endosomes and lysosomes and exhibits a polytopic orientation, forming several luminal and cytoplasmatic loops. NPC1 is a cholesterol transporter and mutations in the *NPC1* gene result in fatal, progressive neurodegenerative disorder, Niemann-Pick C1 disease, due to a defect in the export of cholesterol from lysosomes [186]. The abnormal accumulation of cholesterol in turn leads to altered protein and lipid trafficking [187,188].Which lines of evidence suggest that NPC1 facilitates filovirus entry? NPC1 interacts with primed GP_1_ [20,130] and the contribution of NPC1 to infectious entry of filoviruses can be separated from its cholesterol transport activity [20,129], indicating that the protein directly facilitates entry. NPC1-deficient cells, including primary fibroblasts derived from NPC1 patients, were resistant to filovirus infection, but still allowed for efficient cellular entry of several other viruses [20,129]. Furthermore, transduction of the wild type *NPC1* gene into NPC1-defective, patient-derived cells or NPC1-negative CHO cells fully restored infection [20,129] and directed expression of NPC1 in non-susceptible reptilian cells or haploid hamster CHO-K1 cell clones was sufficient to render these cells susceptible to GP_1,2_-mediated infection [130]. Additionally, siRNA knockdown of NPC-1 in HeLa cells resulted in reduced virus uptake [20]. Finally, heterozygous *NPC1*^−/+^ mice were protected against filovirus infection in sharp contrast to wild type mice [129]. In sum, these results show that NPC1 plays a key role in cellular entry of ebolaviruses.

As the NPC1 protein is localized on the endosomal and lysosomal membranes, it was proposed to act downstream of filovirus GP_1,2_ engagement of attachment and signaling factors at the cell surface. Indeed, GP_1,2_-mediated viral uptake was readily detectable in NPC1-deficient cells, where the virions accumulated in early endosomes, indicating that membrane fusion was not triggered [129]. Proteolytic processing of GP_1,2_ and thus exposure of the RBR was a prerequisite of NPC1 binding, as only the cleaved 19 kDa form was able to physically interact with NPC1 [20,130]. Mapping studies revealed that the 19 kDa form binds to the second luminal domain of NPC1 [130] and cell surface presentation of this domain in the context of an artificial receptor molecule was sufficient to allow entry of pseudotypes carrying thermolysin-primed filovirus GP_1,2_ [130]. It can be speculated that the interaction between the 19 kDa form of GP_1,2_ and NPC1 might expose the GP_2_ residues involved in membrane fusion. However, fusion of pseudotypes bearing the primed 19 kDa GP_1,2_ with the plasma membrane of target cells expressing the second loop of NPC1 at their surface could not be induced by low pH treatment. Thus, binding of primed GP_1,2_ to NPC1 is not sufficient to trigger membrane fusion [130].

Besides filoviruses, flaviviruses have also been found to depend on molecules involved in cholesterol transport for infectious entry into host cells. The cholesterol-transport inhibitor U18666A interfered with hepatitis C and Dengue virus (strain TSV01) infection [189,190], and a factor with 40% amino acid identity to NPC1 (named NPC1-like 1, which is only expressed in human hepatocytes and the intestine) was shown to be involved in uptake of hepatitis C virus [191]. In contrast to filovirus infection, however, hepatitis C virus entry mediated by the NPC1-like 1 protein was dependent on cholesterol uptake and no evidence for an interaction between hepatitis C virus and NPC1-like 1 protein has been reported so far [191].

Taken together, the NPC1 protein is a filovirus receptor with unexpected characteristics: In comparison to cell surface receptors used by other viruses, NPC1 is unique in recognizing the filoviral glycoprotein only after uptake into the cell and after proteolytic processing. The identification of small molecule inhibitors directly or indirectly inhibiting NPC1-usage by GP_1,2_ for host cell entry [20,129] makes it an attractive candidate for antiviral therapy.

## 5. Conclusions

Host cell entry is the first essential step in filovirus infection. Cellular lectins can concentrate vectors bearing GP_1,2_ at the cellular surface and can thereby promote infectious entry. However, lectin expression usually does not render cells susceptible to GP_1,2_-driven entry and a role of lectins in the cell tropism of filoviruses in the infected host remains to be demonstrated. TAM tyrosine kinase receptors and TIM-1 can augment entry of filoviruses into a subset of susceptible cell lines and into some primary cells but are not universally required for filovirus infection. In fact, neither TIM-1 nor the TAM family member Axl are expressed to appreciable amounts in human macrophages, key viral targets, and the factors regulating viral uptake into these cells remain to be elucidated. The respective studies might reveal that filoviruses can use diverse cell surface factors for uptake into TIM-1-, Axl‑negative cells, which might account for the broad cell tropism of filoviruses. In contrast to Axl and TIM-1, the broadly expressed endosomal/lysosomal protein NPC1 is required for filovirus entry into all cellular systems tested so far and seems to play a key role in filovirus entry. Further experiments will clarify whether NPC1 alone is sufficient for triggering membrane fusion or whether another host cell factor is involved. 
